# The Critical Role of *Faecalibacterium prausnitzii* in Cardiovascular Diseases

**DOI:** 10.31083/RCM26740

**Published:** 2025-03-20

**Authors:** Tiantian Zheng, Chenchen Meng, Zhengtian Lv, Chenxia Wu, Xinbin Zhou, Wei Mao

**Affiliations:** ^1^The First School of Clinical Medicine, Zhejiang Chinese Medical University, 310053 Hangzhou, Zhejiang, China; ^2^Department of Cardiology, Affiliated Zhejiang Hospital, Zhejiang University School of Medicine, 310030 Hangzhou, Zhejiang, China; ^3^Department of Cardiology, The First Affiliated Hospital of Zhejiang Chinese Medical University (Zhejiang Provincial Hospital of Chinese Medicine), 310006 Hangzhou, Zhejiang, China; ^4^Zhejiang Key Laboratory of Integrative Chinese and Western Medicine for Diagnosis and Treatment of Circulatory Diseases, 310030 Hangzhou, Zhejiang, China

**Keywords:** gut microbiomes, *Faecalibacterium prausnitzi*, cardiovascular diseases

## Abstract

Due to the continued aging of the global population, cardiovascular diseases (CVDs) remain the main cause of death worldwide, with millions of fatalities from diseases, including stroke and coronary artery disease, reported annually. Thus, novel therapeutic approaches and targets are urgently required for diagnosing and treating CVDs. Recent studies emphasize the vital part of gut microbiota in both CVD prevention and management. Among these, *Faecalibacterium prausnitzii* (*F. prausnitzii*) has emerged as a promising probiotic capable of improving intestinal health. Although preliminary investigations demonstrate that *F. prausnitzii* positively enhances cardiovascular health, research specifically connecting this strain to CVD outcomes remains limited. Based on current research and assessment of possible clinical applications, this paper aimed to investigate the positive effects on cardiovascular health using *F. prausnitzii* and its metabolites. Targeting gut flora is expected to become a mainstay in CVD treatment as research develops.

## 1. Introduction 

Among noncommunicable diseases, cardiovascular disease (CVD) ranks as the main cause of disability and mortality, therefore severely burdening individuals as well as healthcare institutions. With important disorders including coronary heart disease, heart failure (HF), myocardial infarction (MI), and stroke, it is a substantial contributor to global mortality [1, 2, 3]. Good control of CVD calls for an emphasis on early diagnosis, prevention, and sophisticated treatment strategies meant to lower incidence and mortality rates. Recent developments in molecular tools and sequencing technologies including metagenomics and metabolomics are improving our knowledge of the interactions between the host and gut microbiota [4]. This increasing realization emphasizes how gut microbiota and its metabolites preserve intestinal health, regulate inflammation, and influence metabolic processes [5, 6]. Moreover, their significant impact on the evolution of CVD is becoming increasingly apparent [2, 7, 8, 9].

Forming in a complex and interactive ecosystem, the gut hosts a far higher count of microbes than human cells, crucially part of the host’s metabolic system, this microbial community controls intestinal immune responses, help to absorb energy from foods, and maintain metabolic balance [10, 11]. Widely regarded as the largest endocrine organ in the human body, the gut microbiota can generate a range of bioactive compounds that influence many facets of host physiology [12, 13, 14]. Furthermore, interacting with intestinal epithelial cells, the gut flora, a vital component of the physical barrier of the intestinal mucosa, helps to maintain the integrity of the intestinal barrier and support its protective function. Any disturbance in the microbiome can compromise the function of intestinal barrier, thereby influencing the condition of the host [15]. Particularly, we investigate the possible consequences of changes in the composition and function of the gut microbiome on several CVDs, including but not limited to atherosclerosis (AS), thrombosis, HF, and hypertension (HTN) [16, 17, 18, 19, 20].

Among next-generation probiotics (NGPs), *Faecalibacterium prausnitzii (F. prausnitzii)*, a dominant gram-negative bacterium within the Clostridium family of Firmicutes, is a fundamental microorganism linked with gut microbiota imbalances in many diseases, especially inflammatory bowel conditions and gastrointestinal cancers [21, 22, 23, 24, 25]. Maintaining immune balance and promoting gut health depend on its anti-inflammatory and immunological-regulating actions [25, 26, 27, 28, 29]. Furthermore, *F. prausnitzii* produces short-chain fatty acids (SCFAs), particularly butyrate, which is believed to confer benefits for cardiovascular health [26, 30, 31, 32]. It is well established that SCFAs derived from the gut microbiota are causally related to CVDs, including AS, HTN, and HF [4, 10, 33, 34, 35].

While preliminary theories and data suggest that many probiotics may positively influence on cardiovascular health, most studies conducted to date are either descriptive or clinical in nature [3, 36, 37, 38]. There is a limited number of studies specifically linking certain gut microbiota to the prevention of CVD. In this context, we explored the potential effects of *F. prausnitzii* and its metabolites on CVD. This study also highlights the beneficial effects of *F. prausnitzii* on various cardiovascular conditions. These observations suggest that *F. prausnitzii* and its metabolites may serve as valuable gut-based biomarkers for the diagnosis and treatment of CVD. It is anticipated that, in the near future, gut microbiota will emerge as a novel therapeutic target for CVD, alongside potential strategies focused on modulating gut microbial processes.

## 2. Role of *F. prausnitzii* in Physiology and Pathology

*F. prausnitzii*, formerly known as *Fusobacterium prausnitzii*, is a low-guanine-cytosine (GC), gram-positive, non-spore-forming, extremely oxygen-sensitive, non-motile bacterium belonging to the phylum Firmicutes [39]. This species is the predominant strain within the *Clostridium leptum* cluster found in the human colon and is the second most common representative in fecal samples, following *Clostridium coccoides* [40]. *F. prausnitzii* ranks among the most abundant bacteria in the gut microbiota of healthy adults, constituting more than 5% of the total bacterial population. In some individuals, this proportion can increase to approximately 15%. *F. prausnitzii* is commonly present in the gastrointestinal tracts of a variety of mammals, including pigs [41], calves [42], mice [43], and poultry [44]. The abundance and widespread presence of *F. prausnitzii* indicate that it is a crucial component of the microbiota, with potential effects on host physiology and health. Consequently, changes in the abundance of *F. prausnitzii* have been extensively documented in various diseases in humans [21].

*F. prausnitzii* is commonly considered a beneficial probiotic for human metabolism [45]. As a highly metabolically active symbiotic bacterium, it is renowned for its anti-inflammatory properties [26]. This bacterium can ferment glucose to produce various metabolites, including formic acid, D-lactic acid, and butyrate [26, 46]. In contrast, *F. prausnitzii* plays a crucial role in immune regulation by maintaining T-helper 17 (Th17)/regulatory T lymphocyte (Treg) balance, diminishing the production of inflammatory cytokines to suppress inflammatory responses and enhancing gut barrier function. Collectively, these mechanisms collectively contribute to the maintenance of immune balance within the gut [24, 47, 48]. Chronic inflammation is strongly associated with CVD. Therefore, reducing inflammation levels may be crucial for preventing the development and progression of these conditions. As observed in both humans and mice, a decrease in *F. prausnitzii* levels in the gut has been linked to several cardiovascular risk factors, including type 2 diabetes mellitus (T2DM) and obesity [30]. Furthermore, obesity and hyperglycemia are among the cardiovascular risk factors that can disrupt the gut microbiome and cause negative changes directly related with a higher risk of CVD [2, 49, 50]. Moreover, a reduction in *F. prausnitzii* could lead to higher intestinal permeability, therefore raising the chance of endogenous bacterial toxins getting into the bloodstream. Low-grade endotoxemia resulting from this can aggravate chronic inflammatory responses and help CVD to proceed [51]. Recognised as a probiotic with anti-inflammatory and immune-regulating properties is *F. prausnitzii*. Promoting cardiovascular health depends on a balanced gut microbiome, which is maintained in part by suppressing dangerous infections and encouraging microbial diversity [52, 53, 54]. *F. prausnitzii* is therefore an essential component of the gut microbiome that helps to preserve overall health and immune system, and may also have more general physiological impact [27, 30]. Although there is a basic theoretical basis and indirect data supporting this claim, the exact processes and direct clinical evidence on the influence of *F. prausnitzii* on CVD demand further in-depth investigation. Future research should try to clarify the particular processes and therapeutic possibilities related with CVD.

Many studies have shown that butyrate, a main metabolic product of *F. prausnitzii*, is essential for its anti-inflammatory action [22, 28, 55, 56, 57]. Among the most plentiful butyrate-generating bacteria in the gut is *F. prausnitzii* [52]. Butyric acid, a SCFA produced by gut microbial fermentation of dietary fiber is produced for intestinal epithelial cells [32]. It provides energy and also controls immunological responses, shows anti-inflammatory and antioxidant action, and has many advantages for cardiovascular health [58, 59, 60]. For instance, whereas butyrate is a necessary carbon source for colonocytes, it helps to regulate cellular energy by activating protein kinase B (Akt)/mammalian target of rapamycin (mTOR) and modulating adenosine monophosphate (AMP)-activated protein kinase. Tight junction proteins in intestinal epithelial cells are produced and assembled under this control, therefore maintaining the integrity of the gut barrier and preventing higher permeability [33, 61]. Reduced butyrate levels are linked to higher intestinal permeability, which can aggravate CVDs and systemic inflammation [62]. Furthermore, butyrate has a significant impact on overall metabolism by enhancing glucose metabolism and insulin sensitivity, both of which are associated with a reduced risk of CVDs [63]. Studies indicate that butyrate may support cardiovascular health by influencing lipid metabolism, particularly through the reduction of serum triglyceride and cholesterol levels [64]. Furthermore, there is data implying that dysbiosis relates to reduced butyrate generation in different HF cohorts [65]. In cases of HTN, both animal and human studies have shown a decline in bacteria capable of butyrate generation, therefore suggesting a negative correlation between the abundance of butyrate-producing bacteria and blood pressure (BP) [66, 67, 68]. The evidence points to higher sensitivity to developing CVD being associated with a decrease in the abundance of butyrate-producing bacteria, along with downregulation of genes involved for butyrate synthesis, and low butyrate levels overall.

## 3. The Role of *F. prausnitzii* in Cardiovascular Diseases

**Fig. 1.**
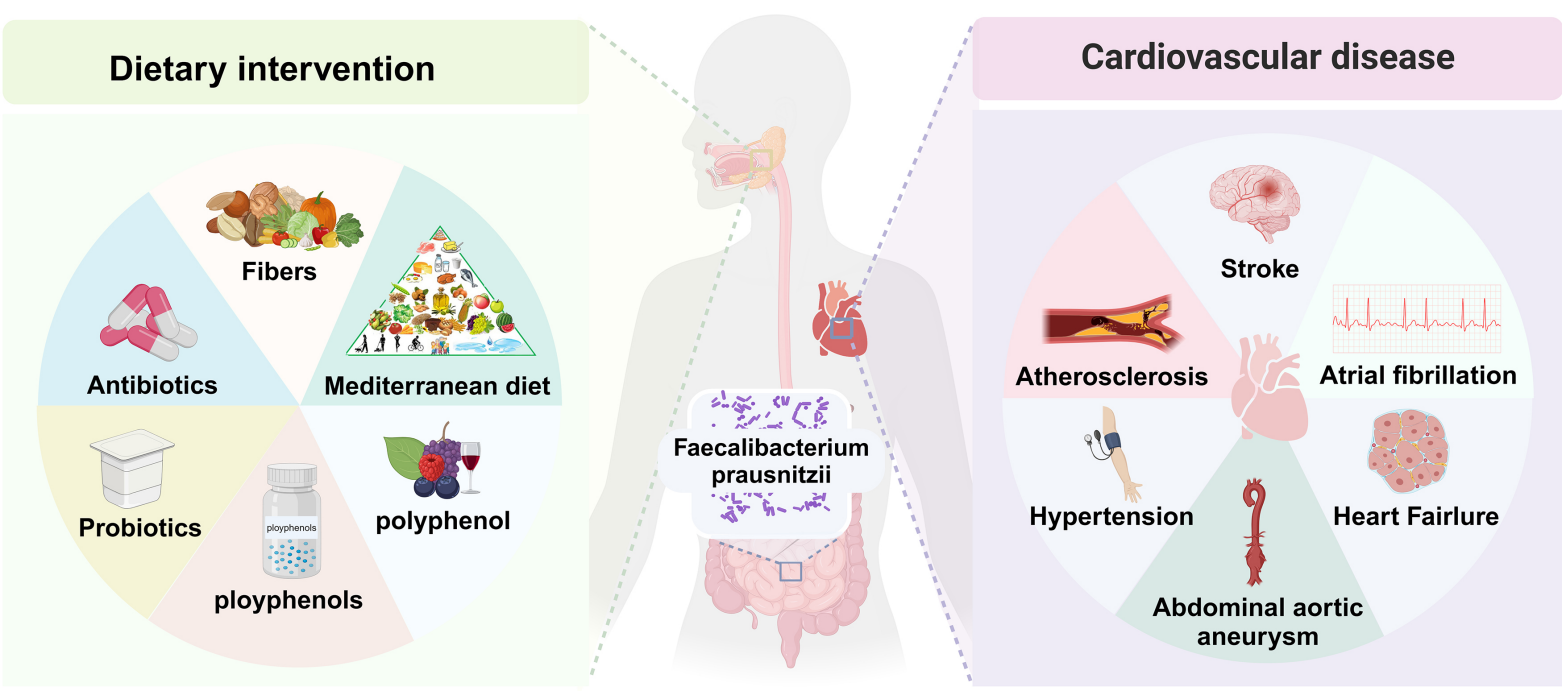
**Intervention factors and potential cardiovascular health of *Faecalibacterium prausnitzii (F. prausnitzii)* in the gut**. By altering dietary patterns to include higher levels of fiber-rich foods, as well as probiotics and prebiotics, it is possible to enhance the abundance of intestinal *F. prausnitzii* bacteria. This modification may contribute to a reduced risk of various cardiovascular diseases (CVDs), including atherosclerosis (AS), stroke, heart failure (HF), atrial fibrillation (AF), and hypertension (HTN).

Recent research has demonstrated that alterations in the composition and structure of gut microbiota, particularly changes in the abundance of *F. prausnitzii*, are associated with the development and progression of various CVDs [30, 69, 70, 71, 72]. *F. prausnitzii* may influence immune and metabolic processes related to cardiovascular health through the gut-heart axis. In this review, we summarize the most recent findings on the interactions between *F. prausnitzii*, its metabolic products, and the mechanisms underlying CVD, including AS, coronary artery disease (CAD), HTN, HF, abdominal aortic aneurysm (AAA), stroke, and atrial fibrillation (AF). Furthermore, we provide a comprehensive overview of the dynamic regulatory effects of this bacterium on CVD (Fig. 1).

### 3.1 Atherosclerosis and Coronary Artery Disease

As the population ages, the prevalence of CAD is increasing. Despite significant advancements in diagnostic and therapeutic techniques, the mortality rate associated with CAD remains high [73]. CAD is defined as myocardial dysfunction and/or structural damage resulting from the narrowing of the coronary arteries and insufficient blood supply. It is categorized into three types based on clinical symptoms, the extent of arterial blockage, and the degree of myocardial damage: stable coronary artery disease (SCAD), unstable angina (UA), and MI [74]. AS is the principal cause of CAD [75]. Recent research has highlighted the role of the gut microbiome, particularly *F. prausnitzii*, in the development and progression of AS and CAD. Investigations examining intestinal microbiota in individuals with various health conditions, particularly CAD, have revealed a significant decrease in *F. prausnitzii* levels [69, 70, 73, 76, 77]. This suggests a potential link between the low abundance of this bacterium and the presence or progression of CAD. Jie *et al*. [78] identified a significant reduction in the levels of *Bacteroides cellulosilyticus*, *F. prausnitzii*, and *Roseburia intestinalis* in individuals with atherosclerotic cardiovascular disease (ACVD), based on a survey of metagenome-wide association studies. Furthermore, Sanchez-Alcoholado *et al*. [76] found that CAD patients with T2DM exhibited significantly reduced levels of beneficial bacteria, such as *F. prausnitzii* and *Bacteroides fragilis*, along with increased levels of opportunistic pathogens, including* Enterobacteriaceae*, *Streptococcus*, and *Desulfovibrio*, compared to CAD patients without T2DM. Furthermore, in the CAD-T2DM group, higher* Enterobacteriaceae* and lower *F. prausnitzii* levels were associated with increased serum trimethylamine N-oxide (TMAO) levels, suggesting that T2DM contributes to impaired immune regulation in CAD through microbial and metabolic changes [76]. Yang *et al*. [51] conducted a clinical cohort study involving 371 participants, comparing patients with CAD to a control group without CAD. The study revealed that individuals with a higher abundance of *F. prausnitzii* exhibited a significantly lower incidence of CAD. Furthermore, random forest modeling further demonstrated a significant negative correlation between *F. prausnitzii* levels and the incidence of CAD. Additionally, the authors found that oral administration of *F. prausnitzii* to *ApoE-/-* mice on a high-fat diet resulted in significant anti-atherosclerotic effects. These effects were associated with improved gut barrier integrity, reduced translocation of intestinal-derived lipopolysaccharide (LPS), and decreased inflammation, rather than increased butyrate production. This indicates that *F. prausnitzii* is a promising candidate for mitigating AS [51].

### 3.2 Hypertension

HTN is a critical and preventable risk factor contributing to the global prevalence of CVD. It imposes a significant economic burden on society and represents a major public health concern [79]. HTN arises from a complex interplay of genetic, environmental, and physiological factors, including disturbances in the vascular system and kidney function [63]. Although the exact mechanisms remain incompletely understood, increasing evidence suggests a substantial link between HTN and imbalances in the gut microbiota [71, 80, 81, 82]. In the study conducted by Yang *et al*. [68], it was reported that spontaneously hypertensive rats, as well as those subjected to long-term Angiotensin (Ang) II injections, exhibited significant decreases in gut microbiota richness, diversity, and evenness compared to the control group. Meanwhile, there was an observed increase in the *Firmicutes/Bacteroidetes* (F/B) ratio of hypertensive rats compared to the control group, indicating an imbalance in the gut microbiota of hypertensive animals [68]. Yan *et al*. [71] investigated the gut microbiome of individuals with HTN by analyzing fecal samples from 60 patients with primary HTN and 60 matched healthy controls (HCs), considering gender, age, and body weight. Using whole-metagenome shotgun sequencing, they discovered that certain bacteria known for producing SCFAs—specifically *Ruminococcaceae*, *Roseburia*, and *Faecalibacterium* species—were found in lower quantities in hypertensive patients compared to those with normal BP [71]. Li *et al*. [80] found that metabolic changes in patients with pre-HTN or HTN were closely associated with dysbiosis of the gut microbiota. By transplanting feces from hypertensive human donors into germ-free mice, they observed an increase in blood pressure, thereby demonstrating the direct impact of the gut microbiota on host blood pressure. Compared with the control group, the abundance of gut microbiota, including *F. prausnitzii*, *Roseburia*, and *Butyrivibrio*, was significantly reduced in patients with HTN [80]. Zheng *et al*. [83] conducted a metagenomic analysis of fecal samples from 30 primary HTN patients undergoing antihypertensive treatment, comparing these samples to those from 8 healthy adults not on any medications. The analysis revealed a significant reduction in *Clostridium leptum*, *F. prausnitzii*, and other strains within the patient group [83]. Despite the observed decrease in the abundance of *F. prausnitzii* among patients with HTN, the underlying mechanisms remain unclear. Future studies aimed at establishing a causal relationship between HTN and *F. prausnitzii* in the gut microbiota could provide valuable insights for the development of new strategies for the treatment and prevention of HTN and related cardiovascular conditions.

### 3.3 Heart Failure

HF is the ultimate consequence of various initial cardiac injuries, compounded by imbalances in compensatory mechanisms and pathological processes. This condition leads to the heart’s inability to efficiently pump blood, thereby failing to meet the physiological demands of the body [84]. The association between HF and alterations in gut function is well established. HF frequently leads to congestion of the visceral circulation, resulting in swelling of the bowel wall and damage to the intestinal barrier. The gut microbiota, which interacts with the cardiovascular system through the gut-heart axis, may promote inflammation and contribute to the progression of HF [85, 86]. Simadibrata *et al*. [87] summarized and included nine peer-reviewed human studies that compared the gut microbiome characteristics of 317 adult patients with HF to those of 510 HCs. The study found that gut microbiome richness and diversity were significantly reduced in patients with HF, accompanied by a notable decrease in SCFA-producing bacteria such as *F. prausnitzii* [87]. Furthermore, the reduction or absence of *F. prausnitzii*, a bacterium essential for regulating inflammation, may exacerbate chronic inflammatory conditions. Kamo* et al*. [88] observed that decreases in *F. prausnitzii* are prevalent among in elderly patients with congestive heart failure (CHF). Considering that inflammation is independently associated with poor outcomes in elderly patients with CHF, it is plausible that an exacerbated inflammatory response contributes to a deteriorating prognosis. This underscores the importance of addressing inflammation in order to improve the outcomes in vulnerable populations. Cui *et al*. [89] found that the composition of the gut microbiota in patients with CHF differed significantly from that in HCs. They identified a decrease in *F. prausnitzii* and an increase in *Ruminococcus gnavus* as key features of the gut microbiota in patients with CHF. Additionally, they observed an imbalance in gut microbes related to the metabolism of beneficial metabolites, such as butyrate, and harmful metabolites, such as TMAO. This reduction in butyrate production may contribute to the exacerbation of chronic inflammation in CHF [89]. Therefore, Addressing the decrease in *F. prausnitzii* and butyrate levels may represent an effective strategy for managing HF.

### 3.4 Abdominal Aortic Aneurysm

AAA typically occurs in the infrarenal portion of the aorta and is described as a segmental, full-thickness dilation of the abdominal aorta, with a diameter exceeding 50% of the normal diameter. It is estimated that approximately 8% of the general population is affected by aortic aneurysms [90]. Unless complications arise, AAA generally presents asymptomatically. The standard treatment approaches include open surgery and endovascular repair, both aimed at effectively addressing the condition [91]. Currently, there are few effective non-invasive treatments available to halt the progression of AAA. Recently, increasing evidence suggests that gut dysbiosis may contribute to the development and progression of AAA [92, 93]. In a systematic review encompassing 12 animal studies and 8 human studies, it was observed that the abundance of *Faecalibacterium* was significantly higher in individuals with AAAs compared to HCs. Furthermore, this increased abundance of *Faecalibacterium* has been associated with larger aneurysms [94]. Xiao *et al*. [95] discovered that in an Ang II-induced AAA mouse model, the fecal abundance of *Prevotella *was greater in the AAA group than in the control group. Previous studies have suggested that *Prevotella* is regarded as a beneficial probiotic, offering metabolic and anti-inflammatory advantages [45]. The authors proposed that the observed increase in *F. prausnitzii* levels might be attributable to a specific pathological subtype of *F. prausnitzii*, either clade A or D [30]. This finding highlights the complexity of interpreting microbiome data in relation to various health conditions and emphasizes the urgent need for further studies to elucidate the role of *Faecalibacterium* in AAA.

### 3.5 Stroke

Stroke is defined as a sudden interruption in blood flow to the brain. There are two primary types: ischemic stroke, which occurs due to clots obstructing cerebral vessels, and hemorrhagic stroke, which results from the rupture of blood vessels within the brain. Ischemic stroke is the most prevalent cause of morbidity and mortality among the elderly population [96]. Although the precise mechanisms remain unclear, growing evidence suggests that stroke can impact gut motility, increase gut permeability, activate resident immune cells in the gut, and lead to a shift in the gut microbiome towards a more harmful state known as dysbiosis [97]. Regardless of the signaling mechanisms underlying the microbiome-gut-brain bidirectional communication, it is widely believed that a positive feedback loop exists. In this loop, alterations in the brain following a stroke lead to gut dysbiosis and inflammation, which in turn exacerbate neuroinflammation post-stroke. This cycle contributes to the progression of stroke [98]. In experimental stroke models, modifying the gut microbiome through techniques such as fecal microbiota transplantation or antibiotic treatment, either prior to or during a stroke, may significantly influence recovery outcomes [99, 100]. Lee *et al*. [72] discovered that fecal transplantation from young donors significantly improved stroke outcomes in older mice. The feces from these young donors were rich in SCFAs and beneficial bacteria. Furthermore, the transplantation of specific SCFAs-producing bacteria, such as *Bifidobacterium longum*, *Clostridium symbiosum*, *F. prausnitzii*, and *Lactobacillus fermentum*, led to reductions in neurological deficits and inflammation following stroke, while simultaneously increasing SCFAs levels in the gut, brain, and blood of older mice. This indicates that SCFAs-producing bacteria, particularly *F. prausnitzii*, may enhance stroke recovery by elevating SCFAs concentrations within the gut-blood-brain axis [72]. Rahman* et al*. [101] found that a synthetic formulation containing multiple probiotics significantly improved post-stroke outcomes in rats subjected to middle cerebral artery occlusion (MCAO). *16S ribosomal RNA* gene sequencing of the gut contents revealed a significant increase in *F. prausnitzii* in the synthetic formulation group compared to the MCAO surgery group [101]. Luo *et al*. [102] conducted a case-control study involving 59 patients with acute ischemic stroke (AIS) and 31 age-matched controls. Their findings revealed a negative correlation between the presence of *F. prausnitzii* and both the severity of stroke and adverse prognostic factors, as well as inflammatory markers. These results suggest that *F. prausnitzii* may play a significant role in the management of AIS [102].

### 3.6 Atrial Fibrillation

AF is the most prevalent form of persistent arrhythmia and represents a significant global health challenge that adversely affects the quality of life for those impacted. Although existing pharmacological treatments can alleviate symptoms to some extent, they are frequently associated with a variety of side effects [103]. Catheter radiofrequency ablation presents a valuable treatment alternative for patients with AF who cannot tolerate antiarrhythmic medications or who experience symptoms that are difficult to manage. Nevertheless, accurately predicting and managing the maintenance of sinus rhythm and the prevention of AF recurrence remain a significant challenge [104]. Consequently, the development of novel and effective predictive models is crucial. Recent preclinical and observational cohort studies have suggested that an imbalance in gut microbiome composition may contribute to the pathogenesis of AF [104]. Zuo *et al*. [105] identified an imbalance in the gut microbiome of AF patients. Specifically, there was an overgrowth of *Ruminococcus* and a significant reduction in *Faecalibacterium*, *Alistipes*, *Oscillibacter*, and *Bilophila*. These microbial changes were evident in both fecal and serum samples, offering potential biomarkers for identifying individuals with AF [105]. In a separate cohort study, Zuo *et al*. [14] found that in patients with persistent AF, regardless of whether the duration was longer than 12 months, the abundance of *Faecalibacterium*, particularly *F. prausnitzii*, was significantly lower compared to the control group. However, this difference may suggest that the probiotic *F. prausnitzii* could play a beneficial role in the pathogenesis of AF, warranting further investigation into its potential effects and mechanisms.

In addition to evidence from AAA studies, a higher abundance of *F. prausnitzii* is generally associated with improved outcomes in CVD, including in CAD, AS, HTN, HF, AF, and stroke. The relevant clinical and basic research results obtained at present are described and summarized, as shown in Table 1 (Ref. [14, 51, 69, 70, 71, 76, 78, 80, 83, 87, 88, 89, 95, 102, 105, 106]) and Table 2 (Ref. [51, 72, 94, 101]) respectively. Consequently, increasing the gut abundance of *F. prausnitzii* through a relatively safe treatment may represent a promising strategy for the prevention or management of CVD. This approach could potentially reduce the incidence of CVD and slow its progression, ultimately benefiting both cardiovascular and overall health.

**Table 1.** **Overview of clinical research of *F. prausnitzii* related to several cardiovascular diseases**.

Cardiovascular disease	Type of study	Experimental subjects	Sample type	Analysis method	Outcome characteristics	Relevance conclusion	Ref.
HP	Human	15 HP, 27 At-HP, 19 controls	Fecal samples	*16S rDNA* Sequencing	↓α, β-diversity; ↑*F. prausnitzii*, ↑*Akkermansia muciniphila*, ↑*Oscillospira*, ↓*Desulfovibrio sp.* in At- HP group compared with HP group	At-HP may selectively restore the abundance of anti-inflammation associated bacteria (including *F. prausnitzii*) that were disrupted in the HP	[69]
						*F. prausnitzii* positively correlated with HDL	
CAD	Human	36 MCS, 91 SA, 48 UA, 66 AMI, 65 controls	Fecal samples	*16S rRNA* Sequencing	↓*Blautia*, ↓*Dorea*, ↓*Tyzzerella*, ↓*Agathobaculum*, ↓*Faecalibacterium* in AMI group	*Faecalibacterium* as a control indicator compared with AMI	[70]
CAD	Human	16 CAD-DM2, 16 CAD-NDM2	Fecal samples	*16S rRNA* Sequencing	↓α, β-diversity; ↑Firmicutes/Bacteroidetes; ↓*F. prausnitzii*, ↓*Bacteroides fragilis*, ↑*Enterobacteriaceae*, ↑*Desulfovibrio* in CAD-DM2 group	*F. prausnitzii* negatively correlated with serum TMAO levels, plasma zonulin and positively associated with serum IL-10 levels	[76]
ACVD	Human	218 ACVD, 187 controls	Fecal samples	*16S rRNA* Sequencing	↓*Bacteroides cellulosilyticus*, ↓*F. prausnitzii*, ↓*Roseburia* in ACVD group	*F. prausnitzii* was negatively correlated with serum levels of TG, LDLC, and CHOL	[78]
CAD	Human	56 SCAD, 106 UA, 53 MI, 56 controls	Fecal samples	Metagenomic sequencing	↓α, β-diversity; ↓*F. prausnitzii* in SCAD, UA, MI	*F. prausnitzii *as a robust independent CAD predictor	[51]
HTN	Human	60 HTN, 60 controls	Fecal samples	Metagenomic sequencing	↓α, β-diversity; ↓*Roseburia spp.*, ↓*F. prausnitzii*, ↑*Klebsiella spp.*, ↑*Streptococcus spp.*, ↑*Parabacteroides merdae* in HTN group	*F. prausnitzii* were less abundant in HTN patients	[71]
HTN	Human	56 pHTN, 99 primary HTN, 41 controls	Fecal samples	Metagenomic sequencing	↓α, β-diversity; ↑*Faecalibacterium*, ↑*Roseburia*, ↑*Butyrivibrio* in controls	*Faecalibacterium* were the common microbial characteristics of pHTN and contributed to the identification of pHTN	[80]
HTN	Human	30 primary HTN patients taking anti-HTN medications, 8 controls	Fecal samples	Metagenomic Sequencing	↓*F. prausnitzii*, ↓*Dorea longicatena*, ↓*Eubacterium hallii*, ↑*Bacteroides fragilis*, ↑*Bacteroides vulgatus*, ↑*Escherichia coli*, ↑*Streptococcus vestibularis* in experimental group	Decrease of *Faecalibacterium* could be common signs of HTN	[83]
HTN	Human	29 non-treated HTN, 32 controls	Fecal samples	*16S rRNA* sequencing, metagenomic analysis	↑*Bacteroides coprocola*, ↑*Bacteroides plebeius*, ↑*Lachnospiraceae*, ↓*Ruminococcaceae NK4A214*, ↓*Ruminococcaceae_UCG-010*, ↓*Christensenellaceae_R-7*, ↓*F. prausnitzii*, ↓*Roseburia hominis* in HT patients	*F. prausnitzii*, as a biomarker of controls group with the highest discriminant power, was negatively correlated with SBP	[106]
HF	Human	317 HF, 510 controls	Fecal samples	*16S rRNA* sequencing	↓α,β-diversity; ↓*Eubacterium rectale*, ↓*F. prausnitzii*, ↓*Oscillibacter sp.*, ↑*Enterococcus*, ↑*Escherichia*, ↑*Klebsiella*, ↑*Lactobacillus*, ↑*Streptococcusin* in HF patients	*F. prausnitzii* were less abundant in HF patients	[87]
HF	Human	12 younger HF, 10 older HF	Fecal samples	*16S rRNA* sequencing	↓*Bacteroidetes*, ↑*Proteobacteria*; ↓*F. prausnitzii*, ↑*Lactobacillus* in older HF	*F. prausnitzii* is associated with worsening inflammation and poor prognosis in HF patients as they age	[88]
HF	Human	53 CHF, 41 controls	Fecal samples	Metagenomic analyses	↓*F. prausnitzii*, ↑*Ruminococcus gnavus* in CHF	*F. prausnitzii* is associated with worsening inflammation and poor prognosis in HF patients as they age	[89]
AAA	Human; animal	12 animal studies; 8 human studies	Fecal samples	*16S rRNA* sequencing	↑*Proteobacteria phylum*, ↑*Campylobacter*, *Fusobacterium*, ↑*F. prausnitzii*, ↓*Akkermansia muciniphila*, ↓*Lactobacillus acidophilus *in AAA group	*F. prausnitzii* were associated with larger aneurysms	[95]
Stroke	Human	59 AIS, 37 controls	Fecal samples	*16S rDNA* sequencing	↓β-diversity; ↓*Faecalibacterium*, ↓*F. prausnitzii* in non-minor stroke and 3-month poor prognosis groups	*F. prausnitzii* is negatively associated with stroke severity, impaired prognosis, and pro-inflammatory markers, highlighting its potential application in AIS treatments	[102]
AF	Human	50 AF, 50 controls	Fecal samples	Metagenomic analyses	↑*Ruminococcus*, ↑*Streptococcus*, ↑*Enterococcus*, ↓*Faecalibacterium*, ↓*Alistipes*, ↓*Oscillibacter*, ↓*Bilophila* in AF group	*F. prausnitzi* was found decreased in the AF group	[105]
AF	Human	12 psAF of <12 months, 8 psAF of >12 months, 20 controls	Fecal samples	Metagenomic sequencing	↓*Faecalibacterium*, particularly ↓*F. prausnitzii* in 12 psAF of <12 months and 8 psAF of >12 months	GM dysbiosis (including *F. prausnitzii*) may contributes to the progression of psAF	[14]

↓, reduce the abundance or concentration; ↑, increase the abundance or concentration; ACVD, atherosclerotic cardiovascular disease; AF, atrial fibrillation; AIS, acute ischemic stroke; AMI, acute myocardial infarction; At-HP, atorvastatin calcium (generic name Lipitor 20 mg dose; Pfizer, Inc., USA)-treated hypercholesterolemic patients; CAD, coronary artery disease; DM2, type-2 diabetes mellitus; CAD-NDM2, CAD patients without DM2; CHF, congestive heart failure; CHOL, Cholesterol; HDL, high density lipoprotein; HF, heart failure; HP, hypercholesterolemic patients; HTN, hypertension; LDLC, low density lipoprotein cholesterol; MCS, mild coronary stenosis; MI, myocardial infarction; pHTN, pre-hypertension; psAF, persistent atrial fibrillation; Ref., reference; SA, stable angina; SBP, systolic blood pressure; SCAD, stable coronary artery disease; UA, unstable angina; TMAO, trimethylamine N-oxide; IL, Interleukin; TG, triglycerides; AAA, abdominal aortic aneurysm; GM, gut microbiota.

**Table 2.** **Overview of basic research of *F. prausnitzii* related to several cardiovascular diseases**.

Cardiovascular disease	Type of study	Model animal	Model animal grouping	Intervention measure	Intervention durations	Outcome characteristics	Relevance conclusion	Ref.
AS	Animal	AS mouse model	*ApoE-/-* mice, n = 20 per group	*F. prausnitzii* (ATCC 27766) solution 2.5 × 10^9^ CFU/100 μL or pbs	oral gavage, 5 times a week for 12 weeks	endotoxaemia: ↑tight junction formation, ↑mechanical and mucosal barriers, ↓intestinal LPS synthesis pathway, ↓plasma LPS; systemic inflammatory response: ↓plasma IL-6, TNF-α, IL-1β; local inflammation: ↓aorta CD68, MCP-1	*F. prausnitzii *has an anti-atherosclerotic effect	[51]
AAA	Animal	AAA mouse model	*ApoE-/-* mice, control group (n = 7), AAA group (n = 13)	Ang II or saline	pump infusion, 4 weeks	↑*Oscillospira*, ↑*Coprococcus*, *F. prausnitzii*, ↑*Alistipes massiliensis*, ↓*Akkermansia muciniphila*, ↓*Allobaculum*, ↓*Barnesiella intestinihominis *in AAA group	*F. prausnitzii *were significantly increased in the AAA group	[94]
Stroke	Animal	experimental stroke mouse model	MCAO aged stroke mice, n = 15 per group	Transplantation of selected SCFA-producing bacterial strains (*Bifidobacterium longum*, *Clostridium symbiosum*, *F. prausnitzii*, *Lactobacillus fermentum*) into a stroke mouse model	oral gavage for 2 days after MCAO	↓poststroke neurological deficits and inflammation; ↑gut, brain and plasma SCFA concentrations in aged stroke mice	SCFAs-producing bacterial strains (including* F. prausnitzii*) can promote poststroke Recovery	[72]
Stroke	Animal	experimental stroke rat model	MCAO rat model, n = 5 per group	new synbiotic formulation containing multistrain probiotics (*Lactobacillus reuteri UBLRu-87*, *Lactobacillus plantarum UBLP-40*, *Lactobacillus rhamnosus UBLR-58*, *Lactobacillus salivarius UBLS-22*, *Bifidobacterium breve UBBr-01*)	oral gavage for 21 days before MCAO	↑poststroke neurological deficits; *16S rRNA* seq: ↑*Prevotella copri*, ↑*Lactobacillus reuteri*, ↑*Roseburia*, ↑*Allobaculum*, ↑*F. prausnitzii*, ↓*Helicobacter*, ↓*Desulfovibrio*, ↓*Akkermansia* in treated group	Multistrain Probiotics Improve MCAO–Driven Neurological Deficits by Revamping Microbiota-Gut-Brain Axis	[101]

↓, reduce the abundance or concentration; ↑, increase the abundance or concentration; AS, atherosclerosis; LPS, lipopolysaccharide; MCAO, middle cerebral artery occlusion; SCFA, short-chain fatty acid; ATCC, American Type Culture Collection; CFU, colony-forming unit; TNF-α, tumor necrosis factor-alpha.

## 4. Conclusions and Future Perspectives

Exploring the Gut Microbiome-Cardiovascular Health Nexus reveals the potential of dietary interventions for CVD Management [107, 108]. Notably, since *F. prausnitzii* plays a beneficial role in the trajectory of CVD progression, it is crucial to scientifically enhance the abundance of this bacterium in the human gut microbiome. Among these, dietary modifications emerge as potent strategies for influencing and modifying the microbiome, serving as a critical primary prevention measure for cardiovascular health [109]. For example, increasing the intake of dietary fiber can be particularly effective. Research indicates that *F. prausnitzii* ferments dietary fiber and higher levels of dietary fiber are positively associated with a greater abundance of this genus in the gut microbiome. Additionally, a fiber-rich Mediterranean diet has been shown to enhance the abundance of this probiotic [110]. Simultaneously, it is advisable to avoid Western diets characterized by high levels of saturated fats, sugars, and processed foods, as this dietary pattern is associated with an increased risk of CVD, obesity, and T2DM. Such diets often result in dysbiosis of the gut microbiome and a significant decrease in the abundance of *F. prausnitzii* [111]. Specifically, a diet high in saturated fat is associated with a reduction in *F. prausnitzii *abundance, while diets rich in monounsaturated and polyunsaturated fatty acids may facilitate its growth [112, 113]. Historically, inulin supplementation has been recognized as one of the earliest and most effective methods for enhancing the abundance of *Faecalibacterium* in the human gut microbiome [114, 115]. Another approach to enhancing the abundance of *F. prausnitzii* involves increasing the intake of prebiotics and polyphenol-rich foods. Moreno-Indias *et al*. [116] found that both healthy individuals and those with metabolic syndrome exhibited increased levels of *F. prausnitzii* following wine consumption. This increase is attributed to the polyphenol prebiotics present in wine [116]. Additionally, the judicious use of antibiotics and other medications is essential to prevent disruptions in the bacterial equilibrium. Maccaferri *et al*. [117] observed that patients treated with rifaximin appropriately experienced an increase in the abundance of *F. prausnitzii* in their microbiomes. In conclusion, by strategically adjusting dietary patterns to enrich the intake of fiber and unsaturated fatty foods, it is feasible to increase the abundance of *F. prausnitzii*, potentially mitigate the risk of CVD [115, 118].

To conclude, substantial epidemiological and experimental evidence strongly supports the consideration of *Faecalibacterium* as a candidate for NGPs or live biotherapeutic products (LBPs) [119]. The beneficial role of *F. prausnitzii* in the development and progression of CVDs positions this bacterium as a promising target for therapeutic intervention through gut microbiome modulation. However, the inclusion of *Faecalibacterium* on the Qualified Presumption of Safety (QPS) list is impeded by its extreme oxygen sensitivity and the absence of a comprehensive safety history. Consequently, a thorough toxicological evaluation and strain characterization are imperative to secure regulatory approval [120]. For *Faecalibacterium* to be effectively integrated into foods, supplements, or pharmaceuticals, it is essential to ensure the strain’s stability and its successful incorporation within the human body. Its clinical efficacy depends on these elements in great part. Moreover, converting therapies depending the gut microbiome into clinical environments calls for a thorough assessment of their safety, efficacy, and any adverse effects inside a strict regulatory framework. This diligence is necessary to guarantee that these approaches may be safely and successfully included into clinical practice, thereby boosting disease management and hence patient outcomes.

Our knowledge of the properties and functions of *F. prausnitzii* is still restricted given the developing situation on the bacterium’s interaction with CVDs. More basic and clinical research is desperately needed to clarify the functional activities of *F. prausnitzii* and look at its possible biomarker value. With continuous research, *F. prausnitzii* is expected to become a vital indicator or therapeutic tool for the diagnosis and treatment of CVD in the near future.

## Abbreviations

AAA, abdominal aortic aneurysm; ACVD, atherosclerotic cardiovascular disease; AF, atrial fibrillation; AIS, acute ischemic stroke; AMI, acute myocardial infarction; At-HP, atorvastatin-treated hypercholesterolemic patients; BP, blood pressure; CAD, coronary artery disease; DM2, type-2 diabetes mellitus; CAD-NDM2, CAD patients without type-2 diabetes mellitus; CVD, cardiovascular disease; CHF, congestive heart failure; CHOL, Cholesterol; HDL, high density lipoprotein; F/B, *Firmicutes/Bacteroidetes*; *F. prausnitzii*, *Faecalibacterium prausnitzii*; HCs, healthy controls; HF, heart failure; HP, hypercholesterolemic patients; HTN, hypertension; LBPs, live biotherapeutic products; LDLC, low density lipoprotein cholesterol; LPS, lipopolysaccharide; MCAO, middle cerebral artery occlusion; MCS, mild coronary stenosis; MI, myocardial infarction; NGPs, next-generation probiotics; pHTN, pre-hypertension; psAF, persistent atrial fibrillation; Ref., reference; SA, stable angina; SBP, systolic blood pressure; SCAD, Stable Coronary Artery Disease; SCFAs, short-chain fatty acids; T2DM, type 2 diabetes mellitus; TMAO, trimethylamine N-oxide; QPS, qualified presumption of safety; UA, unstable angina; Akt, protein kinase B; Ang II, Angiotensin II; ATCC, American Type Culture Collection; CFU, colony-forming unit; GC, guanine-cytosine; GM, gut microbiota; mTOR, mammalian target of rapamycin; TG, triglycerides; Th17, T-helper 17; TNF-α, tumor necrosis factor-alpha; Treg, regulatory T lymphocyte.
